# Clinical Dilemma: Flabby Ridges and the Quest for Aesthetic Denture Fit

**DOI:** 10.7759/cureus.66367

**Published:** 2024-08-07

**Authors:** Aashish R Gupta, Mithilesh M Dhamande, Seema R Kambala, Surekha A Dubey, Ankita Pathak, Shubham U Tawade, Rashi Rathi

**Affiliations:** 1 Prosthodontics, Sharad Pawar Dental College and Hospital, Datta Meghe Institute of Higher Education and Research, Wardha, IND; 2 Prosthodontics and Crown and Bridge, Sharad Pawar Dental College and Hospital, Datta Meghe Institute of Higher Education and Research, Wardha, IND; 3 Conservative Dentistry and Endodontics, Sharad Pawar Dental College, Datta Meghe Institute of Higher Education and Research, Wardha, IND

**Keywords:** kelly syndrome, hobkirk's technique, impression material, neutral zone, flabby ridge

## Abstract

A flabby ridge is a hyperplastic and movable soft tissue that affects both maxillary and mandibular ridges most commonly the maxillary anterior region. This tissue that supports the denture is dynamic and can be dislodged by masticatory pressures, which affects denture stability and causes a loss of peripheral seal. The mobile tissue may be distorted as a result of forces applied during the impression-making process.

For dental practitioners, fabricating dentures on a flabby ridge might be a Herculean endeavor because the dentures may not be stable and may not yield satisfactory outcomes. When treating such patients, practitioners should use extra caution. While treating such circumstances, adjustments to standard impression processes can be helpful. There are several ways to address flabby ridges, such as implant therapy, balanced occlusal load distribution, surgical management, and unique impression techniques. In this case report, a patient with a flabby ridge is treated prosthodontically via an alternative impression approach.

## Introduction

A flabby ridge is a condition characterized by soft, compressible, and fibrous tissue which is typically located in the maxilla’s anterior region due to combination syndrome also known as Kelly syndrome [1]. "Combination syndrome" is a clinical condition that occurs in patients who have edentulous maxilla but have natural teeth remaining in the mandibular anterior region which causes bone loss in the anterior region of the maxilla, hyperplasia in the tuberosity region, and supraeruption of mandibular anterior teeth. The prevalence rate in the edentulous maxilla is approximately 24%, while it is just 5% in the edentate mandible [2]. This tissue that supports the denture may be moved by masticatory pressures, resulting in a loss of the peripheral seal [3]. Individuals with flabby ridge often have poor denture retention, inconsistency or instability of CD during function, and frequent complaints of pain or looseness related to a full denture. Histologically speaking, the flabby tissue is made up of loosely distributed fibers and denser collagenized connective tissue, as well as mucosal hyperplasia [4]. Treatment methods that are used to manage the flabby ridge include surgical and a nonsurgical approach. In the surgical technique, the fibrous or flabby tissue must be surgically removed with a scalpel or by injecting a sclerosing agent before the prosthodontic therapy. The nonsurgical or prosthodontic approach involves different impression techniques using different materials [4]. Ridge resorption continues as individuals mature who are edentulous. Less retention and stability of the denture result from smaller denture bases, which are caused by increased ridge resorption [5]. The neutral zone method is used to construct dentures to solve this issue. In the oral cavity, the neutral zone is the region where the forces applied during functions like swallowing, speaking, and chewing are balanced or neutralized. It is the potential space between the cheeks on one side and the tongue on the other [6]. The aesthetic appearance of a complete denture is dependent as much on the denture base as on the denture teeth being used [7]. The process of improving dentures' aesthetic appeal to make them appear as natural as possible, usually the entire dentures, is referred to as denture characterization. Denture characterization blends technical expertise and artistic ability to produce dentures that are not only practical but also aesthetically beautiful and natural-looking.

This case report presents a streamlined method for managing a resorbed and flabby ridge, and also it includes the characterization of the complete denture to fulfill the patient's needs for both an aesthetic improvement and a functional restoration.

## Case presentation

**Figure 1:**
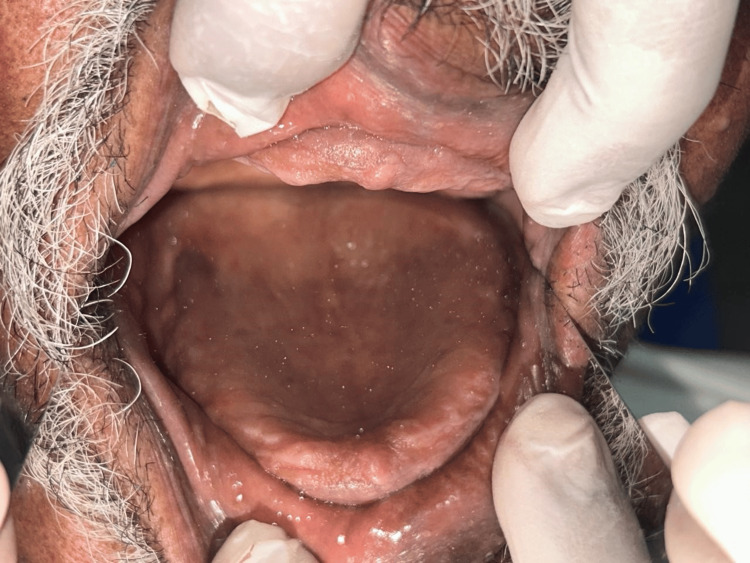
Edentulous maxillary arch

**Figure 2:**
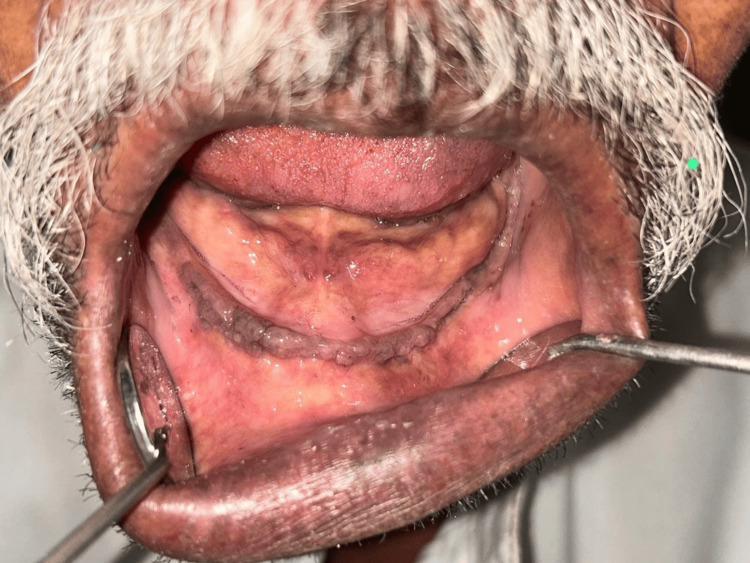
Edentulous mandibular arch

A male patient, who was 61 years old, reported to the Department of Prosthodontics with a chief complaint of loose denture. He stated that he had issues with his speech and pronunciation. He informed that he has been edentulous for the last 10 years and has a habit of supari chewing for 30 years. In addition to being easy to pronounce, the patient requested dentures that resembled the delicate details of tobacco stains and natural coloring. On clinical examination, the mandibular ridge was found to be resorbed and also a flabby ridge from the premolar to the premolar region. A customized treatment plan was designed, taking into consideration the patient's unique demands and specifications (Figures 1-2).

Preliminary Impression

A maxillary preliminary impression was taken using an impression compound and the mandibular impression was taken by irreversible hydrocolloid (alginate). A preliminary cast was poured using type 2 dental stone.

Final Impression

**Figure 3:**
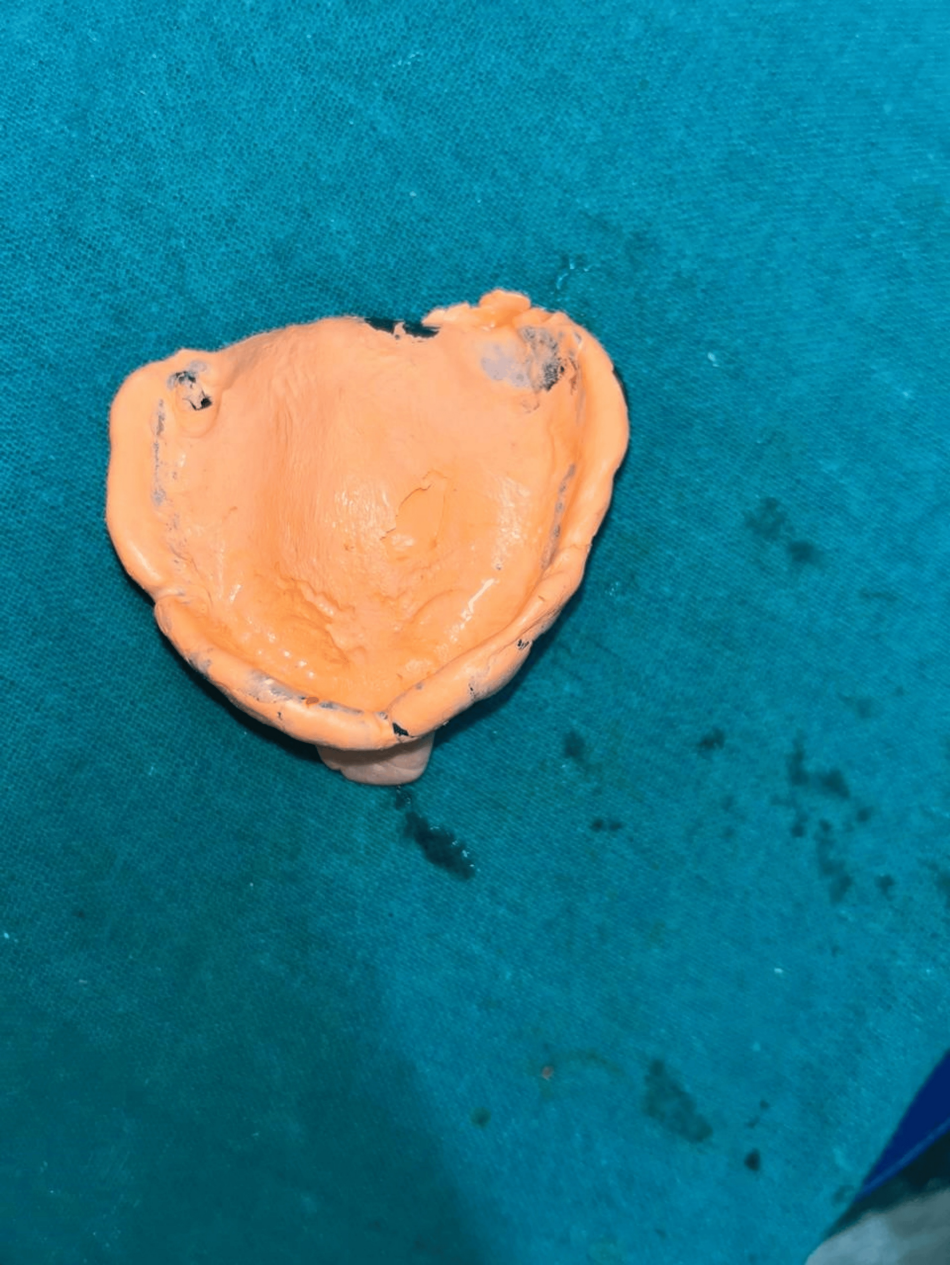
Final impression of the maxilla

**Figure 4:**
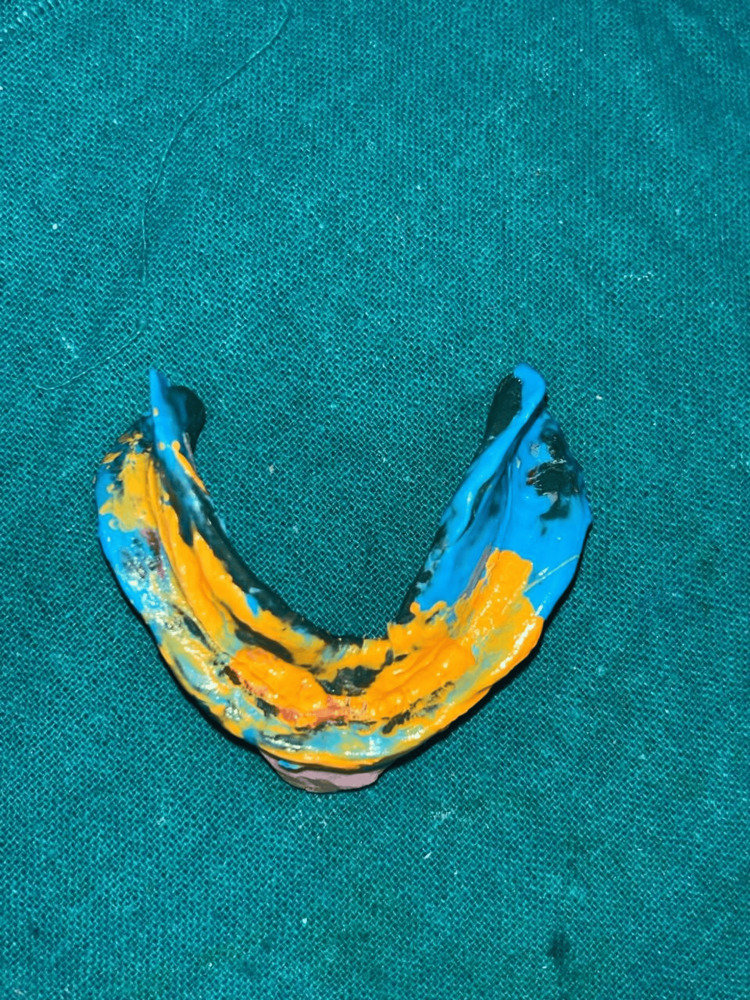
Final impression of the mandible using Hobkirk's technique

A special tray was fabricated using auto-polymerizing polymethyl-methacrylate resin material by providing relief in flabby tissue area with the use of double-spacer design, followed by border molding and the final impression procedure for recording the flabby ridge in an undisplaced position using the Hobkirks technique. Following the appropriate tray extensions being verified, border molding was completed traditionally using green stick impression compound for both maxilla and mandibular. Following border molding, a final impression of the maxilla was made using zinc oxide eugenol paste, and a final impression of the mandible was made using medium body elastomeric impression material. Upon setting of impression material, the tray was taken out of the mouth and the impression material was removed from the area of flabby tissue that is from premolar to premolar. Relief holes were created in the flabby tissue area, and light body elastomeric impression material was poured into the tray to record the flabby tissue. Dental stone was poured into the master cast after that (Figures 3-4).

Jaw Relation and Try-in

**Figure 5:**
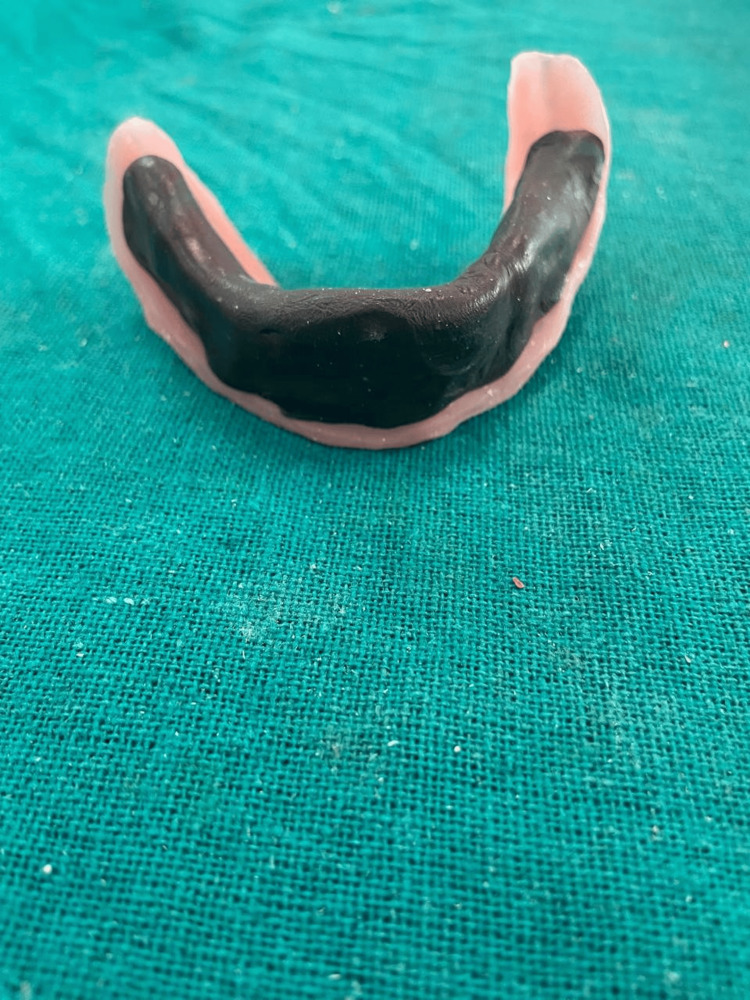
Recorded neutral zone

**Figure 6:**
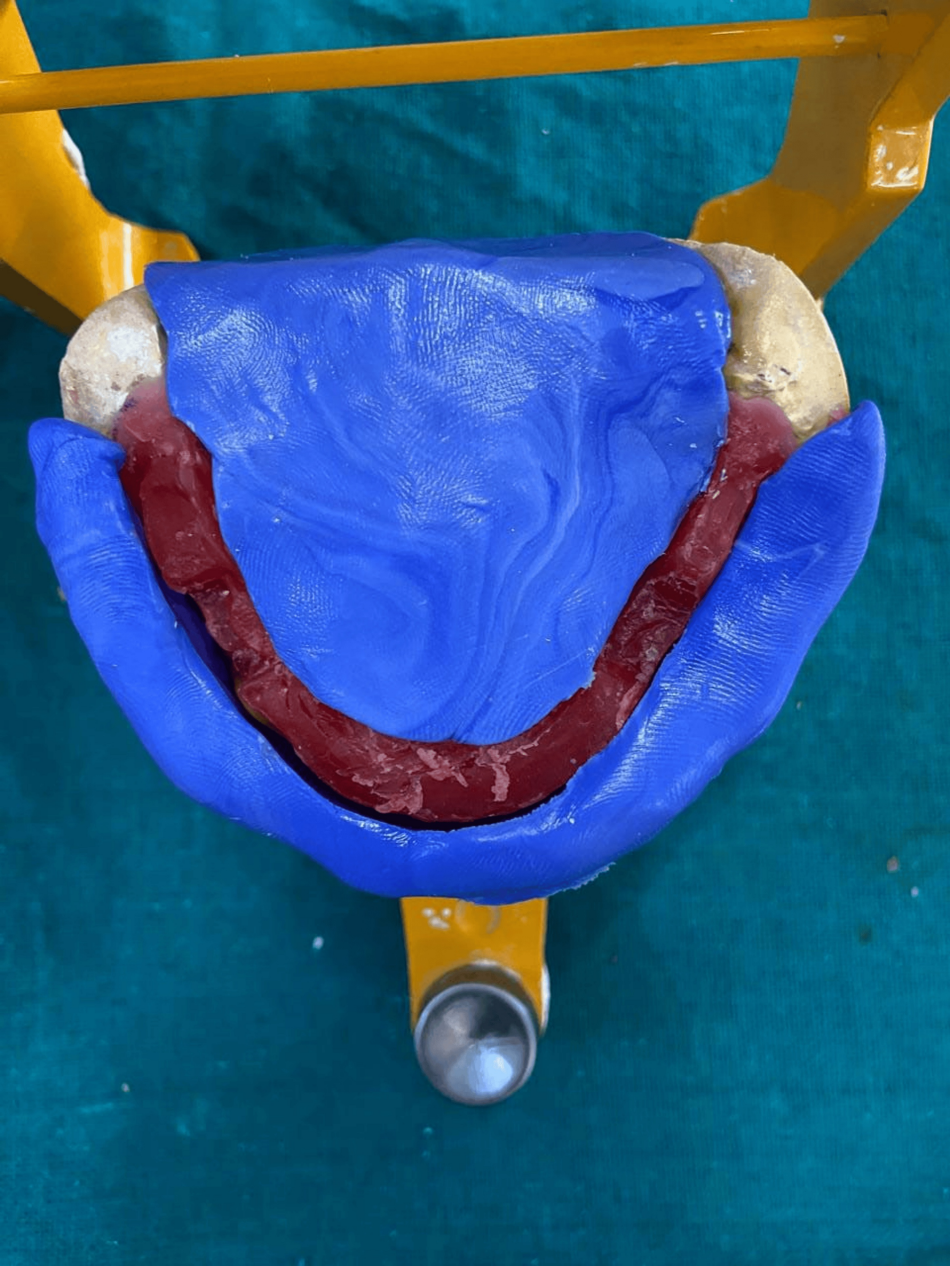
Indexing by putty of neutral zone

Record bases were made on master casts for the mandibular and maxillary arches, after which vertical relation was established and centric relation was recorded. An additional record base was made for the mandibular arch, and orthodontic wire was bent to form loops that would secure the impression material. An admix ratio consisting of three parts green stick compound and seven parts impression compound was used to record the neutral zone which was adapted by softening the material onto the record base with retentive loops to accurately record the neutral zone. The patient was then instructed to do motions including smiling, swallowing, whistling and pronouncing the vowels such as a, e, i, o, u. Following these movements neutral zone was recorded. After this mandibular base plate was removed from the patient's mouth and positioned on the articulator. The neutral zone space was retained with putty-consistent silicone impression material; after that, the impression material was replaced by molten baseplate wax. Followed by teeth arrangement, waxes up, and carving was done for try-in (Figures 5-6).

Fabrication Procedure

**Figure 7:**
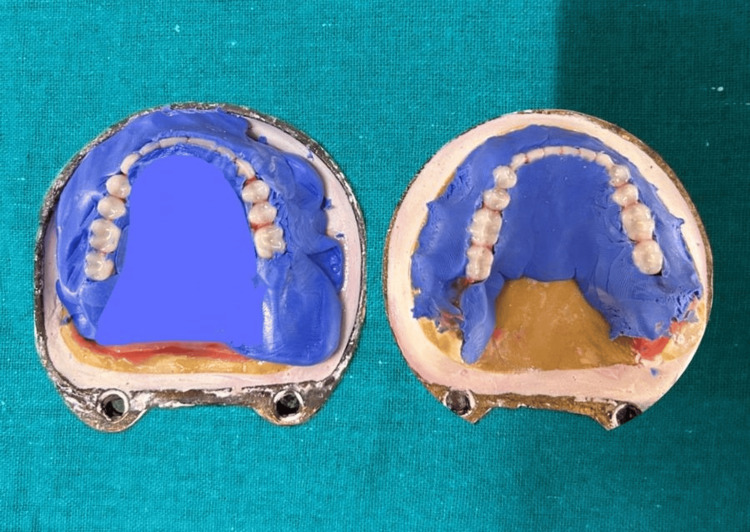
Waxed up denture flasked and adapted with putty silicone

**Figure 8:**
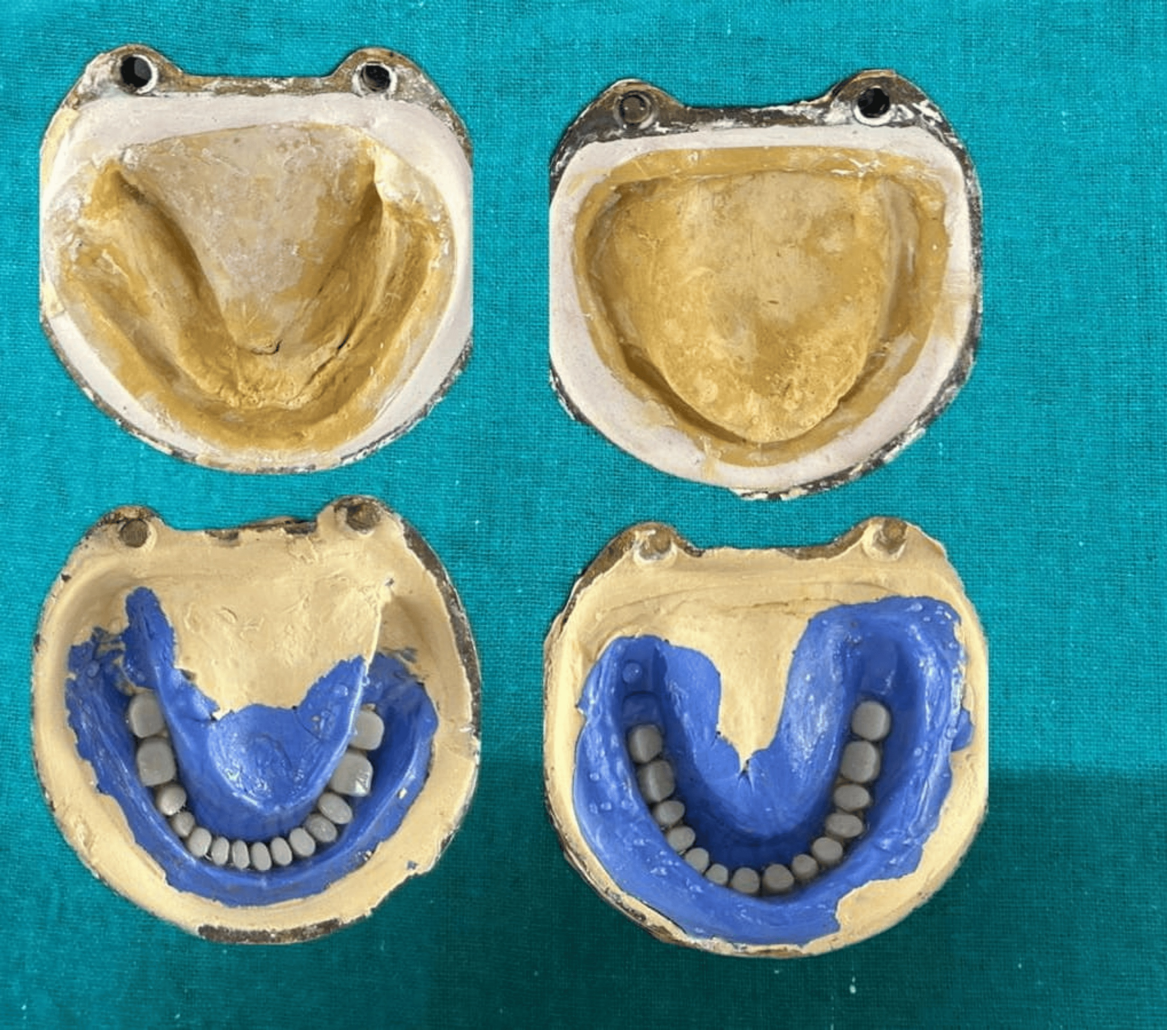
Intrinsic stains added layer by layer on putty

The characterization of dentures was done throughout this process to make them look more natural by mimicking the coloring and tobacco stains. First, the waxed-up denture was invested in a mixture of dental plaster in the first part of the flask after which putty silicone was adapted without incorporating air, the incisal and occlusal surface were slightly exposed from the putty silicone to be held in the investing plaster. After completion of dewaxing the pigments were added layer by layer along the marginal gingiva, interdental papilla followed by light shade pigments were added on the attached gingiva. After that packing and curing were done (Figures 7-8).

**Figure 9:**
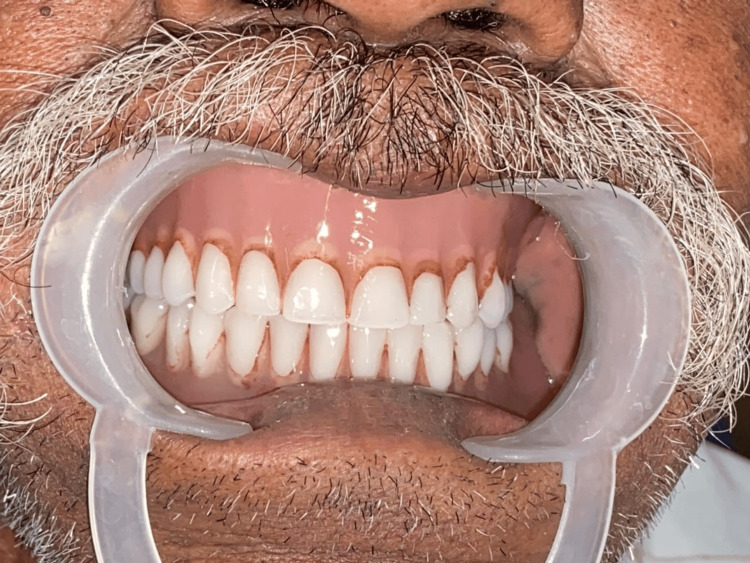
Final prosthesis delivered to the patient incorporated with intrinsic stains

The final denture was delivered to the patient, and the patient was called back for a follow-up appointment of 24 hours and seven days following the denture placement. Follow-up appointments were planned on a regular basis to evaluate retention, function, and aesthetics (Figure 9).

## Discussion

Edentulism affects more than 10% of persons over the age of 50, making it a significant worldwide public health concern [8]. The support of the underlying hard tissue, the bone, and the soft tissue, the mucoperiosteum, are critical to the success of the overall denture prosthesis. Therefore, one of the most crucial determining factors for the effectiveness of wearing complete dentures is the condition and quality of these tissues. A significant body of literature exists regarding treating flabby and resorbed ridges. According to Laskin, if vestibuloplasty is not performed, surgically reducing a flabby ridge covered in scarred mucosa may result in anatomic foundation disturbance [9]. If the tissue is still movable, pendulous, folded, or fissured, and some alveolar bone is still present, surgical reduction by sharp dissection is recommended.

Light body condensation silicone is used in place of impression plaster paint in the Modified William Filler process. Light body imprint material has several benefits, the primary ones being less time-consuming, better flow, less strain on the tissues, and better detail reproduction [9]. Watson suggested a window impression method to reduce the flabby ridge's mobility throughout the function. They created a window in the custom tray above the flabby tissues in the anterior region, and they employed zinc-oxide eugenol impression paste for the healthy denture-bearing area and impression plaster for the flabby ridge. Nevertheless, the window technique's drawback is its inability to apply impression material consistently and under control [10].

There is dynamic muscular activity surrounding the denture. When a denture is built over a heavily resorbed mandibular ridge, the neutral zone approach is used to distribute muscle forces, improve denture retention, and increase stability, which reduces the likelihood that the denture will come loose while being used. The resulting dentures have a better design because there will be less food lodgment and the posterior teeth are positioned appropriately to allow enough space for the tongue [11].

The patient's increased acceptance of the prosthesis is always greatly influenced by its aesthetic appeal. As a natural-looking prosthesis merges harmoniously with the other features of the face, patients seem to value their unique appearance and find it easier to accept [12]. Using novel and distinct methods at every stage, instead of following standard protocols, will maximize the outcomes of full denture creation.

## Conclusions

It can be difficult to treat a patient with a flabby ridge, using typical muco-compressive impression procedures as it is likely to result in an unstable and unretentive denture. These ridges are efficiently manageable by using modified impression processes. It is among the finest substitute methods for treating mandibular ridge atrophy. The primary goal of creating the neutral zone is to create a denture with appropriate muscular balance and a denture with good retention and stability in a patient with resorbed ridges. When compared to a conventional denture, which gives edentulous individuals an unnatural appearance, treatment with a characterized complete denture prosthesis can result in a more lifelike or natural appearance. In addition to helping patients achieve improved speech and appearance, prosthodontic therapy with defined complete denture prostheses can have a significant positive social and psychological impact on patients.
